# Supplementary material for: Dinactin: A New Antitumor Antibiotic with Cell Cycle Progression and Cancer Stemness Inhibiting Activities in Lung Cancer
Source: Antibiotics (Basel). 2022 Dec 19;11(12):1845. doi: 10.3390/antibiotics11121845 (PMC9774622; doi:10.3390/antibiotics11121845)
Supplement: Supplementary file 1 [file antibiotics-11-01845-s001.zip › antibiotics-2098722-supplementary.pdf]

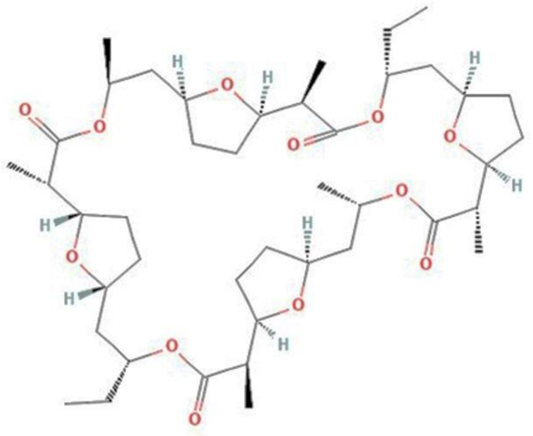

Figure S1. The chemical structure of dinactin.

(National Center for Biotechnology Information (2022). PubChem Compound Summary for CID 6916048, Antibiotic 170t. Retrieved September 14, 2022 from https://pubchem.ncbi.nlm.nih.gov/compound/Antibiotic-170t.

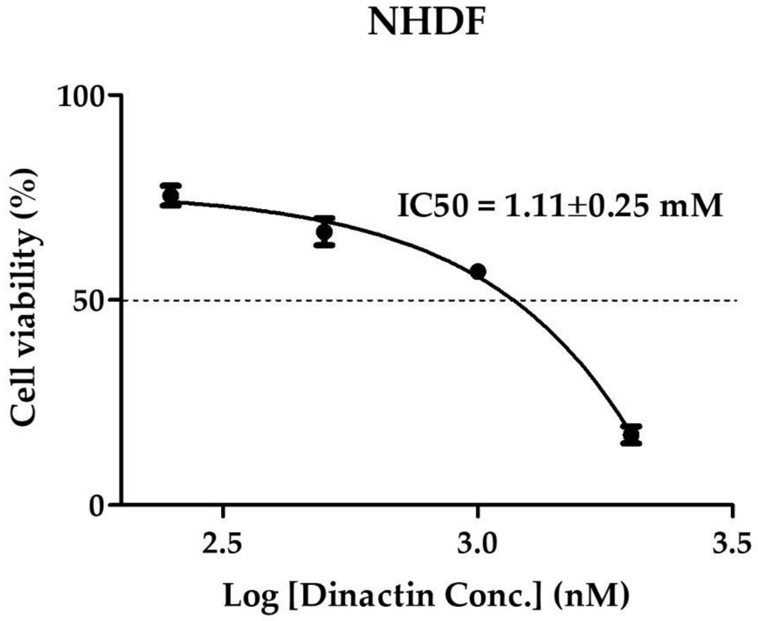

Figure S2. The effect of dinactin on Normal Human Dermal Fibroblast (NHDF) cells.

NHDF cells were treated with various concentrations of dinactin from 0, 0.1, 1, 10, 100, and 1000 nM for 4 days. The IC_{50} values were determined by curve fitting with non-linear regression analysis (sigmoidal dose response). The values were presented as the mean ±SD of three independent experiments.

Table S1. Specifics real-time PCR primers used.

Primer name		5'- nucleotide sequence-3'
ALDH1A1	Forward	GCACGCCAGACTTACCTGTC
	Reverse	CCTCCTCAGTTGCAGGATTAAAG
CD133	Forward	AGTCGGAAACTGGCAGATAGC
	Reverse	GGTAGTGTTGTACTGGGCCAAT
Nanog	Forward	TTTGTGGGCCTGAAGAAAACT
	Reverse	AGGGCTGTCCTGAATAAGCAG
Oct4	Forward	GTGTTCAGCCAAAAGACCATCT
	Reverse	GGCCTGCATGAGGGTTTCT
Sox2	Forward	TACAGCATGTCCTACTCGCAG
	Reverse	GAGGAAGAGGTAACCACAGGG
GAPDH	Forward	TGGTATCGTGGAAGGACTCATGAC
	Reverse	ATGCCACTCAGCTTCCCGTTCAGC

The real-time PCR program used: 95 °C for 5 min, then 45 cycles: 95 °C for 15 sec, melting temperature (Tm) 60 °C for 5 sec, 72 °C for 10 sec. Relative of target mRNA expression was normalized by GAPDH mRNA expression as an internal control. Results were obtained from at least 3 independent experiments.
